# SARS-CoV-2-induced humoral immunity through B cell epitope analysis in COVID-19 infected individuals

**DOI:** 10.1038/s41598-021-85202-9

**Published:** 2021-03-15

**Authors:** Shota Yoshida, Chikako Ono, Hiroki Hayashi, Shinya Fukumoto, Satoshi Shiraishi, Kazunori Tomono, Hisashi Arase, Yoshiharu Matsuura, Hironori Nakagami

**Affiliations:** 1 Department of Health Development and Medicine, Osaka University Graduate School of Medicine, Suita, Japan; 2 Department of Geriatric and General Medicine, Osaka University Graduate School of Medicine, Suita, Japan; 3 Department of Molecular Virology, Research Institute for Microbial Diseases, Osaka University, Suita, Japan; 4 Department of Premier Preventive Medicine, Osaka City University Graduate School of Medicine, Osaka, Japan; 5 Juso Osaka City Hospital, Osaka, Japan; 6 Division of Infection Control and Prevention, Osaka University Hospital, Suita, Japan; 7 Department of Immunochemistry, Research Institute for Microbial Diseases, Osaka University, Suita, Japan; 8 Laboratory of Immunochemistry, WPI Immunology Frontier Research Centre, Osaka University, Suita, Japan

**Keywords:** Immunology, Infection

## Abstract

The aim of this study is to understand adaptive immunity to SARS-CoV-2 through the analysis of B cell epitope and neutralizing activity in coronavirus disease 2019 (COVID-19) patients. We obtained serum from forty-three COVID-19 patients from patients in the intensive care unit of Osaka University Hospital (n = 12) and in Osaka City Juso Hospital (n = 31). Most individuals revealed neutralizing activity against SARS-CoV-2 assessed by a pseudotype virus-neutralizing assay. The antibody production against the spike glycoprotein (S protein) or receptor-binding domain (RBD) of SARS-CoV-2 was elevated, with large individual differences, as assessed by ELISA. We observed the correlation between neutralizing antibody titer and IgG, but not IgM, antibody titer of COVID-19 patients. In the analysis of the predicted the linear B cell epitopes, hot spots in the N-terminal domain of the S protein were observed in the serum from patients in the intensive care unit of Osaka University Hospital. Overall, the analysis of antibody production and B cell epitopes of the S protein from patient serum may provide a novel target for the vaccine development against SARS-CoV-2.

## Introduction

The recent emergence of severe acute respiratory syndrome coronavirus 2 (SARS-CoV-2) and the resulting coronavirus disease 2019 (COVID-19) poses an unprecedented health crisis that was declared a pandemic by the World Health Organization (WHO)^1^. To fight against COVID-19, the rapid development of a vaccine is required in addition to an antiviral drug and an anti-inflammatory drug^2,3^. SARS-CoV-2 belongs to the Betacoronavirus genus, and SARS-CoV-1 and Middle East respiratory syndrome coronavirus (MERS-CoV) are two highly pathogenic viruses in Betacoronavirus genus^4–6^. The spike glycoprotein (S) on the SARS-CoV-2 surface plays an essential role in receptor binding and virus entry, and previous studies on SARS-CoV-1 and MERS-CoV have revealed the importance of the S protein as a potential antigen target for vaccines^7–9^. The S protein has been found to induce robust and protective humoral and cellular immunity, including the development of neutralizing antibodies and T cell-mediated immunity^10–13^.

To understand the immune response to COVID-19, the analysis of virus-specific CD4^+^ and CD8^+^ T cells is required. Grifoni et al*.* recently demonstrated that using HLA class I and II predicted peptide ‘megapools’, circulating SARS-CoV-2-specific CD8^+^ and CD4^+^ T cells were identified in ~ 70% and 100% of COVID-19 convalescent patients, respectively^14^. CD4^+^ T cell responses to S protein were robust and correlated with the magnitude of the anti-SARS-CoV-2 IgG and IgA titers. Importantly, the antibody titer for the receptor-binding domain (RBD) of the S protein correlated well with an increase in spike-specific CD4^+^ T cell responses but not non-Spike-specific CD4^+^ T cell responses. In other reports, RBD-specific antiviral T cell responses have also been detected in people who have recovered from COVID-19^10^.

Here, we addressed the humoral immune response by measuring antibody production against S protein and the neutralizing ability in convalescent patients from two different hospitals. In addition, the B cell epitope of S protein was analyzed by peptide epitope array. These results will assist vaccine design and evaluation of candidate vaccines.

## Results

### Antibody production and neutralizing activity in serum samples from COVID-19 patients

**Figure 1:**
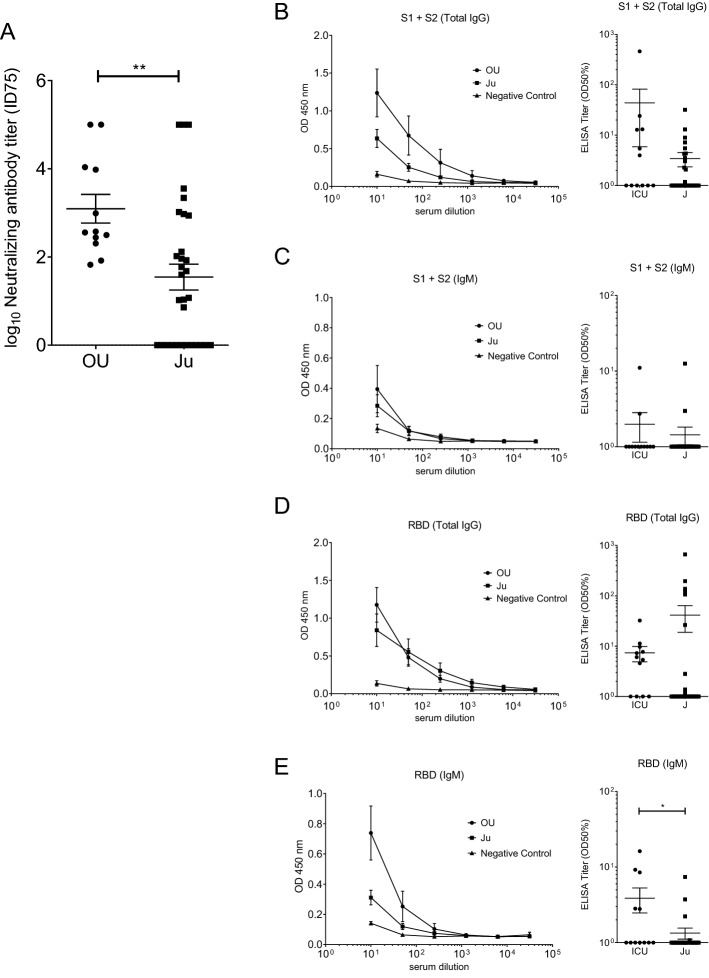
Neutralizing antibody titers and anti-SARS-CoV-2 IgG, IgM responses of COVID-19 patients. (**A**) The neutralizing antibody titers of serum antibodies against SARS-CoV-2 at an evaluation point of the 75% inhibitory dose (ID75). (**B**,**C**) The serum titer against recombinant SARS-CoV-2 spike S1 + S2 protein. (**B**) Total IgG, (**C**) IgM, expressed as the OD at 450 nm and the half-maximal binding (OD 50%). (**D**,**E**) The serum titer against recombinant SARS-CoV-2 spike RBD protein. (**D**) Total IgG, (**E**) IgM, expressed as the OD at 450 nm and the OD 50%. OU, serum samples collected from patients in the ICU of Osaka University Hospital (n = 12); Ju, serum samples collected from patients in Osaka City Juso Hospital (n = 31). All the data are expressed as the mean ± SEM. Statistical evaluation was performed by Mann–Whitney U test (**A**, common logarithmic transformation); *p < 0.05; **p < 0.01. Graphs made in GraphPad Prism version 8.4.3, https://www.graphpad.com/scientific-software/prism/.

To investigate the humoral immunoreaction to SARS-CoV-2, we assessed 43 serum samples collected from COVID-19 patients. Out of 43 patients, 12 patients were in the intensive care unit of Osaka University Hospital (OU samples), and 31 patients were in Osaka City Juso Hospital (Ju samples). To estimate the existence of antibodies against SARS-CoV-2, we performed neutralization tests using pseudotyped vesicular stomatitis viruses (VSVs). At an evaluation point of the 75% inhibitory dose (ID75) (Fig. 1A), we confirmed the average neutralizing activity was higher in samples from Osaka University Hospital (OU) than in samples from Juso Osaka City Hospital (Ju). We speculate that the disease phase and severity of patients may be correlated with these neutralizing activities because most of the patients in Osaka University Hospital are treated in the intensive care unit (ICU) and are more severe than those in Juso Osaka City Hospital.

In the analysis of the humoral response to SARS-CoV-2, we focused on portions of S protein, such as the S1 subunit, S2 subunit, and RBD in the S1 subunit, as candidate antigens. ELISA showed that several sera collected from COVID-19 patients strongly reacted with SARS-CoV-2 recombinant proteins (Table 1 and Supplemental Table 1), and we selected spike S1 + S2 recombinant protein from Beta Lifescience and RBD recombinant protein from Beta Lifescience for further experiments. As shown in Fig. 1B, increased antibody titers to spike (S1 + S2) IgG were observed in about half of the COVID-19 patients. The average antibody titer of the patients in Osaka University Hospital tended to be higher than that of the patients in Juso Osaka City Hospital (*p* = 0.225). The geometric mean titer (GMT) was 4.30 (95% CI: 1.28, 14.46) and 1.84 (95% CI: 1.30, 2.60) in the OU and Ju groups, respectively. Although the difference of IgG antibody levels in both hospitals is not statistically different, we speculate that the disease phase and severity of patients may be also correlated with the total anti-spike IgG titer because most of the patients in Osaka University Hospital were treated in the ICU and were more severe than those in Juso Osaka City Hospital. We additionally analyzed the IgM and IgG subclasses of anti-spike (S1 + S2) antibodies. IgG1 was mainly detected, and IgG3 was less detected (Supplemental Fig. 1A,C). Neither IgG2 nor IgG4 were detected (Supplemental Fig. 1B,D). Compared with the IgG titer, the IgM titer was not so high in all of the patients (Fig. 1C) because all of the samples were obtained from the patients in the recovery phase, not in the acute phase.

**Table 1:** Screening of recombinant proteins for ELISA to measure anti-S1, S2, S1 + S2 or RBD antibodies.

Sample	Sino biological		Beta lifescience				Ray Biotech	
	S1	S2	S1	S2	S1 + S2	RBD	S1	S2
OU #1	3.5*	3.5*	3.5*	2.548	2.664	2.238	2.742	3.5*
OU #2	3.5*	3.5*	3.5*	2.644	2.792	3.286	3.004	3.5*
OU #3	3.5*	3.5*	3.5*	2.461	2.549	2.744	1.263	3.5*
OU #4	0.624	3.5*	0.366	1.262	0.519	0.401	0.186	1.883
OU #5	0.07	3.5*	0.076	0.236	0.177	0.102	0.153	0.427
Negative	0.154	3.5*	0.16	0.124	0.129	0.121	0.265	0.188

OU, serum samples collected from patients in the ICU of Osaka University Hospital.

Negative, negative control serum.

OD at 450 nm, tenfold dilution.

3.5*, over 3.5 Optical Density (calculated as 3.5).

Since the RBD in the S1 subunit is the major target for neutralizing antibodies, we next focused on anti-spike RBD antibodies. Similarly, an increased IgG titer but not IgM titer was observed in most of the samples (Fig. 1D,E). Interestingly, the average anti-spike RBD IgG titer of the patients in Osaka University Hospital are a little low, but not statistically different from that of the patients in Juso Osaka City Hospital (*p* = 0.072), and the GMT was 4.19 (95% CI: 2.00, 8.80) and 2.71 (95% CI: 1.30, 5.66) in the OU and Ju groups, respectively. In terms of IgG subclasses, IgG1 was mainly detected, and IgG3 was less detected (Supplemental Fig. 1E,G), but neither IgG2 nor IgG4 were detected (Supplemental Fig. 1F,H).

**Figure 2:**
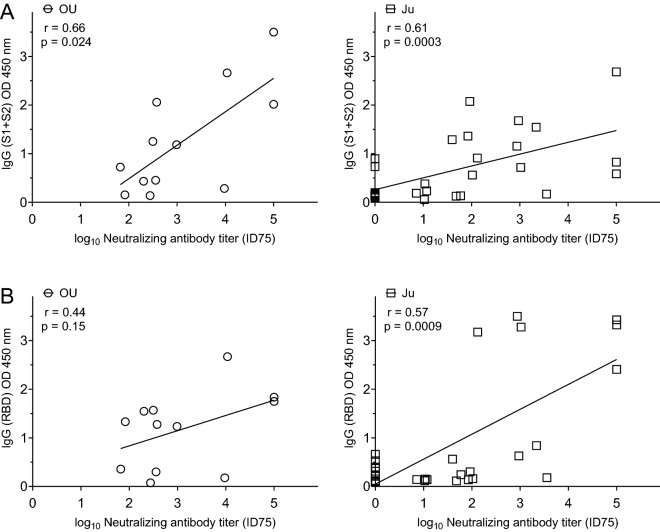
Correlations between neutralizing activity and IgG antibody titer of COVID-19 patients. (**A**) The correlations between neutralizing antibody titer (ID75) and anti-spike S1 + S2 IgG titer (OD 450 nm, tenfold dilution). **(B)** The correlations between neutralizing antibody titer (ID75) and anti-spike RBD IgG titer (OD 450 nm, tenfold dilution). OU (left), serum samples collected from patients in the ICU of Osaka University Hospital (n = 12); Ju (right), serum samples collected from patients in Osaka City Juso Hospital (n = 31). The correlation coefficient was calculated by Spearman’s rank correlation test, common logarithmic transformation (X axes). Graphs made in GraphPad Prism version 8.4.3, https://www.graphpad.com/scientific-software/prism/.

Several publications have reported that neutralizing antibody titer is positively correlated with IgG or IgM antibody binding titer in COVID-19-convalescent individuals^15–17^. We also evaluated the correlation between neutralizing antibody titer and IgG or IgM antibody titer of COVID-19 patients. The correlation between optical density at 450 nm (OD 450 nm) signal for anti-spike (S1 + S2) IgG and neutralizing antibody titer was moderately concordant (Fig. 2A OU, r = 0.66; Ju, r = 0.61). Anti-spike RBD IgG titer (OD 450 nm) also moderately correlated with neutralizing antibody titer (Fig. 2B OU, r = 0.44; Ju, r = 0.57). IgM titer (OD 450 nm) of the anti-spike (S1 + S2) antibody or the anti-spike RBD antibody poorly to moderately correlated with neutralizing antibody titer (Supplemental Fig. 2A,B).

### Analysis of B cell epitopes in S protein in serum samples from COVID-19 patients

**Figure 3:**
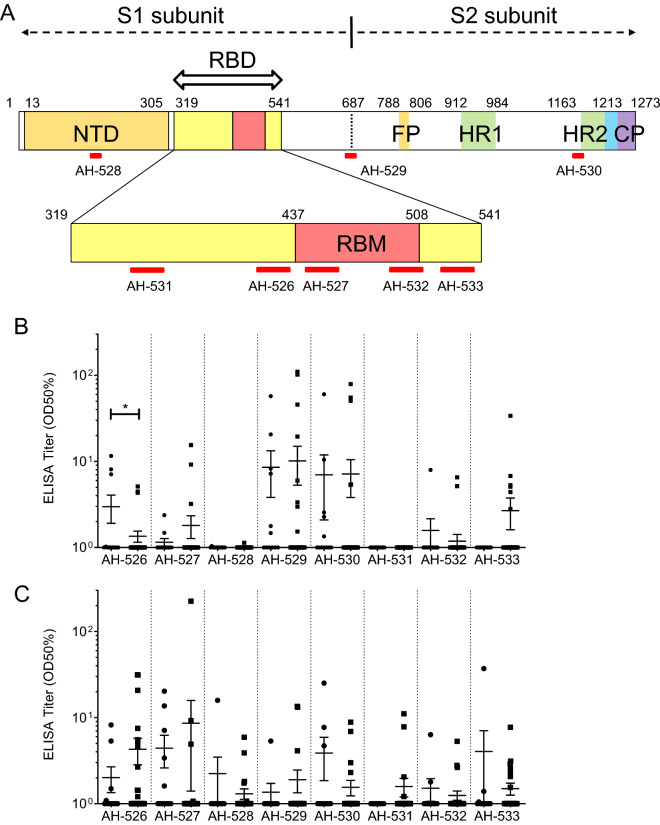
Linear B cell epitopes on Spike protein of SARS-CoV-2 determined by ELISA. (**A**) Scheme of predicted linear B cell epitopes in the RBD or other regions of Spike protein in SARS-CoV-2. As shown in red bars, five regions (AH-531, 346–365 aa.; AH-526, 413–432 aa.; AH-527, 442–460 aa.; AH-532, 491–509 aa.; and AH-533, 518–537 aa.) were selected from the RBD, and three regions (AH-528, 146–164 aa.; AH-529, 671–690 aa.; and AH-530, 1146–1164 aa.) were selected from the NTD, S1 and S2 subunits. NTD, N-terminal domain; RBD, receptor-binding domain; RBM, receptor-binding motif; FP, fusion peptide; HR1, heptad repeat 1; HR2, heptad repeat 2; CP, cytoplasm domain. (**B**,**C**) The serum titer against synthetic SARS-CoV-2 peptides (AH-526 to AH-533) is expressed as the half-maximal binding (OD 50%). (**B**) Total IgG and (**C**) IgM. closed circle, serum samples collected from patients in the ICU of Osaka University Hospital (n = 12); closed square, serum samples collected from patients in Osaka City Juso Hospital (n = 31). All the data are expressed as the mean ± SEM. Statistical evaluation was performed by Mann–Whitney U test; *p < 0.05. Graphs made in GraphPad Prism version 8.4.3. https://www.graphpad.com/scientific-software/prism/.

Furthermore, we predicted linear B cell epitopes by BepiPred-2.0, a prediction tool of B cell epitopes assessed by the characterization of amino acid sequences. Five regions (AH-531, 346–365 aa.; AH-526, 413–432 aa.; AH-527, 442–460 aa.; AH-532, 491–509 aa.; and AH-533, 518–537 aa.) were selected from the RBD, and three regions (AH-528, 146–164 aa.; AH-529, 671–690 aa.; and AH-530, 1146–1164 aa.) were selected from other regions (Fig. 3A and Table 2). In the analysis of IgG (Fig. 3B), two regions, AH-529 (including the S1/S2 cleavage site) and AH-530 (heptad repeat 2, HR2), strongly reacted with sera from some of the patients. Four regions of the RBD (AH-526, AH-527, AH-532 and AH-533) mildly reacted with sera from some of the patients, and the other regions, AH-528 (N-terminal domain, NTD) and AH-531 (RBD), did not react. In the analysis of IgM (Fig. 3C), in contrast, four regions (AH-526, AH-527, AH-529, and AH-530) and the other four regions mildly reacted with sera from some of the patients.

**Table 2:** Amino acid sequence of each synthetic SARS-CoV-2 peptide.

Peptide ID	Position	Amino acid sequence
AH-526	413–432	G–Q–T–G–K–I–A–D–Y–N–Y–K–L–P–D–D–F–T–G–C
AH-527	442–460	D–S–K–V–G–G–N–Y–N–Y–L–Y–R–L–F–R–K–S–N
AH-528	146–164	H–K–N–N–K–S–W–M–E–S–E–F–R–V–Y–S–S–A–N
AH-529	671–690	C–A–S–Y–Q–T–Q–T–N–S–P–R–R–A–R–S–V–A–S–Q
AH-530	1146–1164	D–S–F–K–E–E–L–D–K–Y–F–K–N–H–T–S–P–D–V
AH-531	346–365	R–F–A–S–V–Y–A–W–N–R–K–R–I–S–N–C–V–A–D–Y
AH-532	491–509	P–L–Q–S–Y–G–F–Q–P–T–N–G–V–G–Y–Q–P–Y–R
AH-533	518–537	L–H–A–P–A–T–V–C–G–P–K–K–S–T–N–L–V–K–N–K

**Figure 4:**
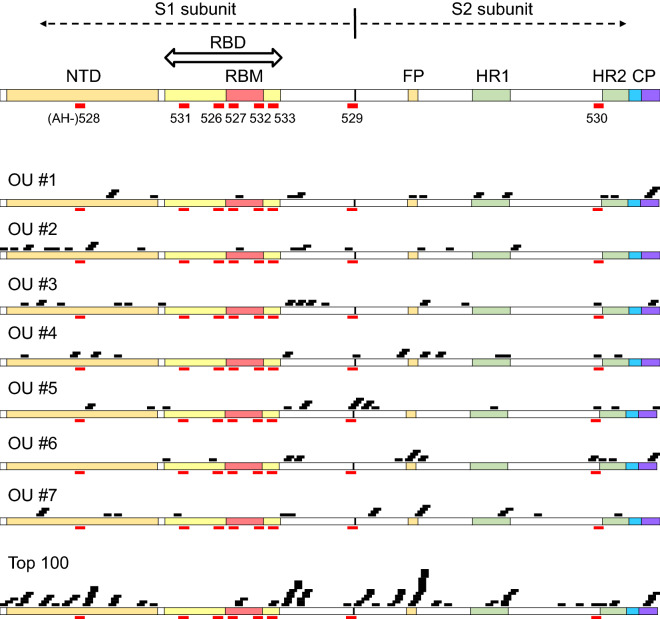
Mapping of the top 20 strongest binding peptide regions of serum samples. The scheme of lists and maps of the top 20 peptide regions with high intensity values for each individual (OU #1-#7). A series of 15-mer peptides overlapping by five amino acids (i.e., 1–15 aa., 5–20 aa., 10–25 aa., etc.) were displayed in this peptide array. The black block indicates the top 20 strongest binding peptide regions with serum samples from each individual and the top 100 strongest binding peptide regions for all samples. The red bars show the candidate linear B cell epitopes: five regions from the RBD (AH-531, 346–365 aa.; AH-526, 413–432 aa.; AH-527, 442–460 aa.; AH-532, 491–509 aa.; and AH-533, 518–537 aa.) and three regions from the NTD, S1 and S2 subunits (AH-528, 146–164 aa.; AH-529, 671–690 aa.; and AH-530, 1146–1164 aa.). NTD, N-terminal domain; RBD, receptor-binding domain; RBM, receptor-binding motif; FP, fusion peptide; HR1, heptad repeat 1; HR2, heptad repeat 2; CP, cytoplasm domain. Data analyzed with Image Lab Software (version 6.0.1, https://www.bio-rad.com/en-jp/product/image-lab-software?ID=KRE6P5E8Z).

We further evaluated the linear B cell epitope within spike protein using a CelluSpots peptide array composed of a series of 15-mer peptides overlapping by five amino acids (i.e., 1–15 aa., 5–20 aa., 10–25 aa., etc.). The lists and maps of the top 20 peptides with high intensity values for each sample (Fig. 4 and Table 3) show that a large number of strongly binding B cell epitopes were located in the regions outside the RBD, such as the NTD, fusion peptide (FP), HR2 and cytoplasm domain (CP). For instance, several strongly binding epitopes in samples OU #1, #2, and #6 were located in the CP, NTD, and FP, respectively. Moreover, we evaluated the linear B cell epitope within nucleocapsid, membrane and envelope proteins using a CelluSpots peptide array. As shown in Supplemental Table 2, consistent with previous findings^18^, most of the strongly binding B cell epitopes were located in nucleocapsid protein. Strong binding antibodies were also detected against nucleocapsid protein by ELISA (Supplemental Fig. 3A and Supplemental Table 3). In addition, the serum samples of non-COVID-19 patients collected from Osaka City University Hospital in 2019 were hardly cross-reacted with nucleocapsid protein and were not cross-reacted with spike (S1 + S2) protein and spike RBD protein (Supplementary Fig. 3A,B).

**Table 3:** Top 20 strongest binding peptide regions of serum samples from each individual (OU #1–7).

#1	Epitope ID	Position	Amino acid sequence
1	K12	1256–1270	F–D–E–D–D–S–E–P–V–L–K–G–V–K–L
2	K11	1251–1265	G–S–C–C–K–F–D–E–D–D–S–E–P–V–L
3	I 3	971–985	G–A–I–S–S–V–L–N–D–I–L–S–R–L–D
4	J17	1161–1175	S–P–D–V–D–L–G–D–I–S–G–I–N–A–S
5	B20	216–230	L–P–Q–G–F–S–A–L–E–P–L–V–D–L–P
6	K10	1246–1260	G–C–C–S–C–G–S–C–C–K–F–D–E–D–D
7	E19	571–585	D–T–T–D–A–V–R––D–P–Q–T–L–E–I–L
8	D20	456–470	F–R–K–S–N–L–K–P–F–E–R–D–I–S–T
9	E20	576–590	V–R–D–P–Q–T–L–E–I–L–D–I–T–P–C
10	G19	811–825	K–P–S–K–R–S–F–I–E–D–L–L–F–N–K
11	I 4	976–990	V–L–N–D–I–L–S–R–L–D–K–V–E–A–E
12	C11	291–305	C–A–L–D–P–L–S–E–T–K–C–T–L–K–S
13	B19	211–225	N–L–V–R–D–L–P–Q–G–F–S–A–L–E–P
14	E16	556–570	N–K–K–F–L–P–F–Q–Q–F–G–R–D–I–A
15	B18	206–220	K–H–T–P–I–N–L–V–R–D–L–P–Q–G–F
16	G15	791–805	T–P–P–I–K–D–F–G–G–F–N–F–S–Q–I
17	H17	921–935	K–L–I–A–N–Q–F–N–S–A–I–G–K–I–Q
18	J21	1181–1195	K–E–I–D–R–L–N–E–V–A–K–N–L–N–E
19	H16	916–930	L–Y–E–N–Q–K–L–I–A–N–Q–F–N–S–A
20	K13	1261–1275	S–E–P–V–L–K–G–V–K–L–H–Y–T

#2	Epitope ID	Position	Amino acid sequence
1	B11	171–185	V–S–Q–P–F–L–M–D–L–E–G–K–Q–G–N
2	B12	176–190	L–M–D–L–E–G–K–Q–G–N–F–K–N–L–R
3	A21	101–115	I–R–G–W–I–F–G–T–T–L–D–S–K–T–Q
4	A10	46–60	S–V–L–H–S–T–Q–D–L–F–L–P–F–F–S
5	G18	806–820	L–P–D–P–S–K–P–S–K–R–S–F–I–E–D
6	A 1	1–15	M–F–V–F–L–V–L–L–P–L–V–S–S–Q–C
7	A 5	21–35	R–T–Q–L–P–P–A–Y–T–N–S–F–T–R–G
8	B 2	126–140	V–V–I–K–V–C–E–F–Q–F–C–N–D–P–F
9	I 7	991–1005	V–Q–I–D–R–L–I–T–G–R–L–Q–S–L–Q
10	F14	666–680	I–G–A–G–I–C–A–S–Y–Q–T–Q–T–N–S
11	I 6	986–1000	K–V–E–A–E–V–Q–I–D–R–L–I–T–G–R
12	A11	51–65	T–Q–D–L–F–L–P–F–F–S–N–V–T–W–F
13	E20	576–590	V–R–D–P–Q–T–L–E–I–L–D–I–T–P–C
14	E17	561–575	P–F–Q–Q–F–G–R–D–I–A–D–T–T–D–A
15	D20	456–470	F–R–K–S–N–L–K–P–F–E–R–D–I–S–T
16	B10	166–180	C–T–F–E–Y–V–S–Q–P–F–L–M–D–L–E
17	E22	586–600	D–I–T–P–C–S–F–G–G–V–S–V–I–T–P
18	H 5	861–875	L–P–P–L–L–T–D–E–M–I–A–Q–Y–T–S
19	C 6	266–280	Y–V–G–Y–L–Q–P–R–T–F–L–L–K–Y–N
20	A18	86–100	F–N–D–G–V–Y–F–A–S–T–E–K–S–N–I

#3	Epitope ID	Position	Amino acid sequence
1	E20	576–590	V–R–D–P–Q–T–L–E–I–L–D–I–T–P–C
2	G20	816–830	S–F–I–E–D–L–L–F–N–K–V–T–L–A–D
3	G19	811–825	K–P–S–K–R–S–F–I–E–D–L–L–F–N–K
4	A 9	41–55	K–V–F–R–S–S–V–L–H–S–T–Q–D–L–F
5	C14	306–320	F–T–V–E–K–G–I–Y–Q–T–S–N–F–R–V
6	J14	1146–1160	D–S–F–K–E–E–L–D–K–Y–F–K–N–H–T
7	K12	1256–1270	F–D–E–D–D–S–E–P–V–L–K–G–V–K–L
8	E16	556–570	N–K–K–F–L–P–F–Q–Q–F–G–R–D–I–A
9	E24	596–610	S–V–I–T–P–G–T–N–T–S–N–Q–V–A–V
10	A16	76–90	T–K–R–F–D–N–P–V–L–P–F–N–D–G–V
11	E19	571–585	D–T–T–D–A–V–R–D–P–Q–T–L–E–I–L
12	A23	111–125	D–S–K–T–Q–S–L–L–I–V–N–N–A–T–N
13	E15	551–565	V–L–T–E–S–N–K–K–F–L–P–F–Q–Q–F
14	E23	591–605	S–F–G–G–V–S–V–I–T–P–G–T–N–T–S
15	A15	71–85	S–G–T–N–G–T–K–R–F–D–N–P–V–L–P
16	F 5	621–635	P–V–A–I–H–A–D–Q–L–T–P–T–W–R–V
17	K11	1251–1265	G–S–C–C–K–F–D–E–D–D–S–E–P–V–L
18	B21	221–235	S–A–L–E–P–L–V–D–L–P–I–G–I–N–I
19	C 1	241–255	L–L–A–L–H–R–S–Y–L–T–P–G–D–S–S
20	H11	891–905	G–A–A–L–Q–I–P–F–A–M–Q–M–A–Y–R

#4	Epitope ID	Position	Amino acid sequence
1	B21	221–235	S–A–L–E–P–L–V–D–L–P–I–G–I–N–I
2	B 5	141–155	L–G–V–Y–Y–H–K–N–N–K–S–W–M–E–S
3	B12	176–190	L–M–D–L–E–G–K–Q–G–N–F–K–N–L–R
4	E15	551–565	V–L–T–E–S–N–K–K–F–L–P–F–Q–Q–F
5	A 9	41–55	K–V–F–R–S–S–V–L–H–S–T–Q–D–L–F
6	G11	771–785	A–V–E–Q–D–K–N–T–Q–E–V–F–A–Q–V
7	I 3	971–985	G–A–I–S–S–V–L–N–D–I–L–S–R–L–D
8	E14	546–560	L–T–G–T–G–V–L–T–E–S–N–K–K–F–L
9	G10	766–780	A–L–T–G–I–A–V–E–Q–D–K–N–T–Q–E
10	G19	811–825	K–P–S–K–R–S–F–I–E–D–L–L–F–N–K
11	B 4	136–150	C–N–D–P–F–L–G–V–Y–Y–H–K–N–N–K
12	G20	816–830	S–F–I–E–D–L–L–F–N–K–V–T–L–A–D
13	H 1	841–855	L–G–D–I–A–A–R–D–L–I–C–A–Q–K–F
14	J14	1146–1160	D–S–F–K–E–E–L–D–K–Y–F–K–N–H–T
15	J22	1186–1200	L–N–E–V–A–K–N–L–N–E–S–L–I–D–L
16	B13	181–195	G–K–Q–G–N–F–K–N–L–R–E–F–V–F–K
17	G12	776–790	K–N–T–Q–E–V–F–A–Q–V–K–Q–I–Y–K
18	H 2	846–860	A–R–D–L–I–C–A–Q–K–F–N–G–L–T–V
19	H24	956–970	A–Q–A–L–N–T–L–V–K–Q–L–S–S–N–F
20	F17	681–695	P–R–R–A–R–S–V–A–S–Q–S–I–I–A–Y

#5	Epitope ID	Position	Amino acid sequence
1	F22	706–720	A–Y–S–N–N–S–I–A–I–P–T–N–F–T–I
2	E23	591–605	S–F–G–G–V–S–V–I–T–P–G–T–N–T–S
3	B11	171–185	V–S–Q–P–F–L–M–D–L–E–G–K–Q–G–N
4	F23	711–725	S–I–A–I–P–T–N–F–T–I–S–V–T–T–E
5	E22	586–600	D–I–T–P–C–S–F–G–G–V–S–V–I–T–P
6	F18	686–700	S–V–A–S–Q–S–I–I–A–Y–T–M–S–L–G
7	F19	691–705	S–I–I–A–Y–T–M–S–L–G–A–E–N–S–V
8	F21	701–715	A–E–N–S–V–A–Y–S–N–N–S–I–A–I–P
9	B10	166–180	C–T–F–E–Y–V–S–Q–P–F–L–M–D–L–E
10	J22	1186–1200	L–N–E–V–A–K–N–L–N–E–S–L–I–D–L
11	H23	951–965	V–V–N–Q–N–A–Q–A–L–N–T–L–V–K–Q
12	C16	316–330	S–N–F–R–V–Q–P–T–E–S–I–V–R–F–P
13	F17	681–695	P–R–R–A–R–S–V–A–S–Q–S–I–I–A–Y
14	J15	1151–1165	E–L–D–K–Y–F–K–N–H–T–S–P–D–V–D
15	C10	286–300	T–D–A–V–D–C–A–L–D–P–L–S–E–T–K
16	G 1	721–735	S–V–T–T–E–I–L–P–V–S–M–T–K–T–S
17	E15	551–565	V–L–T–E–S–N–K–K–F–L–P–F–Q–Q–F
18	F16	676–690	T–Q–T–N–S–P–R–R–A–R–S–V–A–S–Q
19	K14	1266–1280	E–P–V–L–K–G–V–K–L–H–Y–T
20	E21	581–595	T–L–E–I–L–D–I–T–P–C–S–F–G–G–V

#6	Epitope ID	Position	Amino acid sequence
1	G17	801–815	N–F–S–Q–I–L–P–D–P–S–K–P–S–K–R
2	G15	791–805	T–P–P–I–K–D–FG–G–F–N–F–S–Q–I
3	G19	811–825	K–P–S–K–R–S–F–I–E–D–L–L–F–N–K
4	G20	816–830	S–F–I–E–D–L–L–F–N–K–V–T–L–A–D
5	E15	551–565	V–L–T–E–S–N–K–K–F–L–P–F–Q–Q–F
6	K12	1256–1270	F–D–E–D–D–S–E–P–V–L–K–G–V–K–L
7	G16	796–810	D–F–G–G–F–N–F–S–Q–I–L–P–D–P–S
8	E19	571–585	D–T–T–D–A–V–R–D–P–Q–T–L––E–I–L
9	E16	556–570	N–K–K–F–L–P–F–Q–Q–F–G–R–D–I–A
10	G10	766–780	A–L–T–G–I–A–V–E–Q–D–K–N–T–Q–E
11	J14	1146–1160	D–S–F–K–E–E–L–D–K–Y–F–K–N–H–T
12	E20	576–590	V–R–D–P–Q–T–L–E–I–L–D–I–T–P–C
13	J21	1181–1195	K–E–I–D–R–L–N–E–V––A–K–N–L–N–E
14	K14	1266–1280	E–P–V–L–K–G–V–K–L–H–Y–T
15	K13	1261–1275	S–E–P–V–L–K–G–V–K–L–H–Y–T
16	D10	406–420	E–V–R–Q–I–A–P–G–Q–T–G–K–I–A–D
17	J17	1161–1175	S–P–D–V–D–L–G–D–I–S–G–I–N–A–S
18	J13	1141–1155	L–Q–P–E–L–D–S–F–K–E–E–L–D–K–Y
19	G14	786–800	K–Q–I–Y–K–T–P–P–I–K–D–F–G–G–F
20	C16	316–330	S–N–F–R–V–Q–P–T–E–S–I–V–R–F–P

#7	Epitope ID	Position	Amino acid sequence
1	G19	811–825	K–P–S–K–R–S–F–I–E–D–L–L–F–N–K
2	A16	76–90	T–K–R–F–D–N–P–V–L–P–F–N–D–G–V
3	F23	711–725	S–I–A–I–P–T–N–F–T–I–S–V–T–T–E
4	G 1	721–735	S–V–T–T–E–I–L–P–V–S–M–T–K–T–S
5	G18	806–820	L–P–D–P–S–K–P–S–K–R–S–F–I–E–D
6	F24	716–730	T–N–F–T–I–S–V–T–T–E–I–L–P–V–S
7	A17	81–95	N–P–V–L–P–F–N–D–G–V–Y–F–A–S–T
8	G20	816–830	S–F–I–E–D–L–L–F–N–K–V–T–L–A–D
9	G17	801–815	N–F–S–Q–I–L–P–D–P–S–K–P–S–K–R
10	I 4	976–990	V–L–N–D–I–L–S–R–L–D–K–V–E–A–E
11	E13	541–555	F–N–F–N–G–L–T–G–T–G–V–L–T–E–S
12	A15	71–85	S–G–T–N–G–T–K–R–F–D–N–P–V–L–P
13	B21	221–235	S–A–L–E–P–L–V–D–L–P–I–G–I–N–I
14	I 2	966–980	L–S–S–N–F–G–A–I–S–S–V–L–N–D–I
15	J22	1186–1200	L–N–E–V–A–K–N–L–N–E–S–L–I–D–L
16	I15	1031–1045	E–C–V–L–G–Q–S–K–R–V–D–F–C–G–K
17	I 1	961–975	T–L–V–K–Q–L–S–S–N–F–G–A–I–S–S
18	C20	336–350	C–P–F–G–E–V–F–N–A–T–R–F–A–S–V
19	E16	556–570	N–K–K–F–L–P–F–Q–Q–F–G–R–D–I–A
20	B17	201–215	F–K–I–Y–S–K–H–T–P–I–N–L–V–R–D

## Discussion

Here, we report a screening and validation of predicted B cell epitopes of SARS-CoV-2 utilizing human serum from convalescing COVID-19 patients. Several publications have reported about the IgG or IgM antibody in COVID-19-convalescent individuals^15,19,20^. We focused on S protein, especially on the RBD, because it has been reported that anti-RBD antibodies correlate well with an increase in spike-specific CD4^+^ T cell responses.

In the present study, patient sera were obtained from two different hospitals. Osaka University Hospital primarily admits severe patients requiring the ICU, and patient status might be in the subacute phase. Juso Osaka City Hospital, in contrast, usually admits mild or moderate patients, and patient status might be in the convalescent phase. Interestingly, the average antibody titer to S protein was higher in samples from Osaka University Hospital, which was consistent with the high titers in severe patients. Of importance, several patients possessed neutralizing activity with a high titer of IgG for S protein, which may suggest the functional importance of IgG for S protein as neutralizing antibodies. In addition, based on previous findings^14^, these results also suggest that the antibody titer to the RBD of S protein may predict an increase in spike-specific CD4^+^ T cell responses.

Antibodies targeting the RBD and S protein have enhanced potential for providing cross-protective immunity^21^. The bioinformatics approach has been rapidly reported to identify potential B and T cell epitopes in S protein, which has provided data regarding antigen presentation, antibody-binding properties, predicted evolution of epitopes^21–26^, and interaction with immune sensors^27–29^. A list of glycoprotein amino acid positions having a high probability of predicted B cell epitopes has been compiled. Based on the location of the relevant amino acid positions in the model structure, several epitope regions were predicted, i.e., 491–505 aa. and 558–562 aa. in the RBD, and the calculated surface of the amino acid residues of B cell epitopes are shown, i.e., 491–505 aa. and 558–562 aa. in the RBD and 1140–1146 aa. in other regions. We also performed predictions for linear B cell epitopes by BepiPred 2.0, the characterization of amino acids and the predicted structure. Seven regions (346–365 aa., 413–432 aa., 442–460 aa., 491–509 aa., and 518–537 aa. in the RBD and 671–690 aa. and 1146–1164 aa. in other regions) were prepared as B cell epitopes, which partially overlapped with previous reports^21–26^. In this study, antibodies against the RBD were not evident, but only a few antibodies recognized these B cell epitopes in the RBD with high neutralizing activity. These results indicate that the RBD region of S protein is not highly immunogenic, and the other neutralizing antibodies beside the RBD region may be involved in individuals with COVID-19.

The detailed analysis of antibody production by peptide array for S protein showed us possible candidate antigens in addition to the RBD. A few individuals (#2, #3, and #4) possessed neutralizing antibodies and showed several strongly binding epitopes in the NTD of S protein. Interestingly, Chi et al*.* recently reported that a neutralizing human antibody binds to the NTD of S protein of SARS-CoV-2 but does not block the interaction between ACE2 and S protein^30^. In their structural model, the monoclonal antibody interacts with the five loops for the NTD, especially between N3 (141–156 aa.) and N5 (246–260 aa.), and three glycosylation sites (Asn17, Asn61, and Asn149) were identified in this structure. Interestingly, as shown in Table 3, several epitopes in #2, #3, and #4 overlapped these regions in the NTD. Although our predicted epitope in the NTD (AH-528; 146–164 aa) did not react with the sera from the individuals in this study, we speculate that the NTD in S protein may be another candidate region for neutralizing antibodies.

As a study limitation, this study protocol has been approved to analyze only human serum samples without any clinical information. Because the onset of infection or severity of patients cannot be known, we cannot discuss the time course of antibodies with the clinical status of the patients. Although the magnitude of IgG production might be dependent on the duration of COVID-19, we can evaluate the dominant B cell epitope of each patient. There have been concerns regarding vaccine enhancement of disease by certain candidate COVID-19 vaccine approaches via antibody-dependent enhancement (ADE). This phenomenon is observed when non-neutralizing virus-specific IgG facilitates entry of virus particles into Fc-receptor-expressing cells, leading to inflammatory activation of macrophages and monocytes^31^. A study in SARS-CoV-1-infected *rhesus macaques* found that anti-S IgG contributes to severe acute lung injury and massive accumulation of monocytes/macrophages in the lung^32^. Thus, the possibility of ADE may have to be considered even though some pre-clinical studies using a SARS-CoV-2 vaccine did not show any evidence of ADE^2,33,34^. The analysis of B-cell epitope of anti-spike antibody may be required in the analysis with the correlation to the neutralizing activity. Although the size of our study is not enough to predict the good epitope candidate of B-cell epitope, the candidate B-cell epitope might be important to develop the safe COVID-19 vaccine. Thus, in the next generation we will try to ideal the epitope vaccine to specifically induce the neutralizing antibody by utilizing the B-cell epitope. To realize this novel vaccine, follicular helper T-cell epitope, which are essential for B cell production of high-affinity, class-switched antibodies, should be also analyzed in combination with HLA (human leukocyte antigen) typing for SARS-CoV-2. Although a few vaccines for COVID-19 has been already approved and injected in the world, this type of novel epitope vaccine might be useful as a booster vaccine to induce the neutralizing antibody production through the activation of memory T-cell and B-cell.

In summary, we conducted full B cell epitope mapping and validated the predicted B cell epitope of S protein, utilizing human sera from patients with COVID-19. Based on the analysis of neutralizing activity, anti-S antibodies might be correlated with the neutralizing action of the antibodies. The results may provide a novel target for the vaccine development against SARS-CoV-2.

## Methods

### Production of pseudotyped VSVs with S protein and transfection experiments

The pseudotype vesicular stomatitis viruses (VSVs) was prepared as described previously^35,36^. Pseudotyped VSVs and recombinant VSVs in which the G gene is replaced by a foreign reporter gene, such as luciferase, were generated. Either 293 T or BHK (Baby Hamster Kidney fibroblasts) cells were grown to 90% confluence on 35-mm tissue culture plates. The cells were infected with a recombinant vaccinia virus encoding the bacteriophage T7 RNA polymerase (vTF7-3) at a multiplicity of infection (MOI) of 5. After incubation at room temperature for 1 h, the cells were transfected with helper plasmids, pBS-N, pBS-P, pBS-L, and pBS-G, and template plasmids, pVSVΔG–Luci, using a cationic liposome reagent. After 4 h, the supernatants were replaced with 10% FBS DMEM, and the cells were incubated at 37 °C for 48 h. The supernatants were then filtered through a 0.22-μm pore-size filter to remove vaccinia virus and were applied to 293 T or BHK cells that had been transfected with pCAGVSVG 24 h previously. Recovery of the virus was assessed by examining the cells for the cytopathic effects that are typical of a VSV infection after 24 h. Stocks of G-complemented viruses, i.e., VSVΔG virus or recombinant viruses transiently bearing VSV G protein on the virion surface, were grown from a single plaque on BHK cells transfected with pCAGVSVG and then stored at − 80 °C. The infectious titers of the recovered viruses were determined by a plaque assay. To generate pseudotype virus, 293 T, BHK, or some other type of cells that exhibit a high competency of transfection were transfected with a plasmid expressing the envelope protein using a cationic liposome reagent. After 24 h of incubation at 37 °C, cells were infected at an MOI of 0.5 with G-complemented-VSVΔG–Luci. The virus was adsorbed for 2 h at 37 °C and then extensively washed four times or more with serum-free Dulbecco’s Modified Eagle’s Medium (DMEM; Nacalai Tesque, Kyoto, Japan). After 24 h of incubation at 37 °C, the culture supernatants were collected, centrifuged to remove cell debris, and stored at − 80 °C. To generate pseudotype VSVs bearing the SARS-CoV-2 S protein, we transfected an expression plasmid encoding SARS-CoV-2 S protein. Pseudotyped particles were harvested 24 h post-inoculation and clarified from cellular debris by centrifugation.

### Cell lines and culture conditions

VeroE6/TMPRSS2 (Transmembrane protease, serine 2) cells, obtained from the JCRB cell bank in Japan, were maintained in DMEM (Nacalai Tesque, Kyoto, Japan) supplemented with 10% (v/v) heat-inactivated FBS and 1 mg/ml Geneticin (G418; Nacalai Tesque, Kyoto, Japan). The cell lines were incubated at 37 °C and 5% CO2.

### Serum samples

The serum samples of COVID-19 patients were obtained from Osaka University Hospital and Osaka City Juso Hospital. Control serum was obtained from pooled human serum (#BJ11787, Tennessee Blood Services, TN, USA) mixed from five males and five females, which were collected in February 2019 and confirmed to be non-infected. Additionally, the serum samples of non-COVID-19 patients were obtained from the MedCity21 health examination registry of Osaka City University Hospital Advanced Medical Center for Preventive Medicine (Osaka, Japan), which were also collected in 2019.

### Pseudotyped virus neutralization assay

For the neutralization assay, VeroE6/TMPRSS2 cells were seeded with a concentration at 1 × 10^5^ cells/ml in a volume of 100 μl on 96-well plates and were incubated in DMEM supplemented with 2% (v/v) heat-inactivated FBS and without G418 at 37 °C and 5% CO2 for 24 h. After 24 h incubation, 10 μl of a 1:1 mixture of pseudotyped virus solution (5 × 10^5^ focus-forming units/ml) and diluted sera from COVID-19 patients was added to VeroE6/TMPRSS2 cells, and then incubated at 37 °C and 5% CO2 for more 24 h. The sera from COVID-19 patients were serially diluted from 10- to 100,000-fold in FBS-free DMEM. The 1:1 mixture was pre-incubated at 37 °C for 1 h before added to VeroE6/TMPRSS2 cells. After incubation, washing each well with PBS, each of the cells was completely lysed with 50 μl of Cell Culture Lysis Reagent (Promega), and then 10 μl of lysed solution was transferred to 96 well white microplates (Berthold Technologies, Germany). Finally, the relative light units (RLUs) 10 s after 40 μl of Luciferase Assay Substrate Solution (Promega) added were measured using a CentroXS3 LB960 (Berthold Technologies) and analyzed with MikroWin 2010 version 5.22 (Berthold Technologies). The 75% inhibitory dose (ID75) was defined as the serum dilution at which the RLUs were reduced by 75% compared with the non-infected control serum wells. The ID75 values were analyzed with non-linear regression (log [inhibitor] vs. response [four parameters]) using GraphPad Prism version 8.4.3 for windows (GraphPad Software).

### Synthetic SARS-CoV-2 peptides

Based on high antigenicity analysis of the three-dimensional predicted structure and B cell epitope information (BepiPred-2.0), eight different antigenic peptides were selected from the amino acid sequence of SARS-CoV-2 (Fig. 3A and Table 2). The synthetic peptide was purified by reverse-phase HPLC (> 98% purity) (Peptide Institute Inc., Osaka, Japan.). The synthetic SARS-CoV-2 peptide was reconstituted at 5 mg/ml in sterile PBS and stored below − 20 °C.

### Enzyme-linked immunosorbent assay

SARS-CoV-2 recombinant proteins, such as Recombinant 2019-nCoV Spike S1 + S2 Protein (ECD; Beta Lifescience), Recombinant 2019-nCoV Spike Protein (RBD; Beta Lifescience), Recombinant SARS-CoV-2 (COVID-19) Nucleocapsid Protein (Acro Biosystems) and other recombinant spike proteins (Table 1), were diluted in 50 mM carbonate buffer to a concentration of 1 μg/ml and coated at 50 ng/well on 96-well ELISA plates (MaxiSorp Nunc, Thermo Fisher Scientific K.K., Japan) overnight at 4 °C. Synthetic SARS-CoV-2 peptides (Table 2; Peptide Institute Inc., Osaka, Japan) were diluted to a concentration of 10 μg/ml and coated at 500 ng/well. After blocking with 100 μl of sheep serum (16,070–096, Gibco), the sera from COVID-19 patients were serially diluted from 10- to 31,250-fold in blocking buffer, added to each well, and incubated overnight at 4 °C. After washing each well with 0.05% PBS Tween-20 (PBS-T), the cells were incubated with horseradish peroxidase (HRP)-conjugated antibodies specific for human IgG (1:10,000; AP004, Binding Site, U.K.) or IgM (1:5,000; AP012, Binding Site) with PBS containing 5% skim milk for 3 h at room temperature. For the IgG subclass determination assay, anti-human IgG subclass-specific HRP-conjugated antibodies (1:10,000; IgG1 (AP006), IgG2 (AP007), IgG3 (AP008), and IgG4 (AP009), Binding Site) were used. After washing the wells with PBS-T, color was developed with the peroxidase chromogenic substrate 3,3′,5,5′-tetramethylbenzidine (TMB; Sigma Aldrich, MO, USA), and the reactions were terminated with 0.9 N sulfuric acid. The absorbance was measured at 450 nm using an iMark Microplate Absorbance Reader (Bio-Rad, CA, USA) and analyzed with MPM6 version 6.1 (Bio-Rad). The half-maximal antibody titer was analyzed with non-linear regression (sigmoidal, 4PL, X is log [concentration]) using GraphPad Prism version 8.4.3 and was determined according to the highest value in the dilution range of each sample.

### Peptide array

CelluSpots peptide array was performed using CelluSpots covid19_hullB (98.301, Intavis) or CelluSpots covid19_hullS (98.302, Intavis) according to the manufacturers’ instructions: blocked by immersing the slides in PBS containing 5% skim milk overnight at 4 °C on an orbital shaker, incubated with diluted serum samples (1:10) overnight at 4 °C on an orbital shaker, washed with PBS-T buffer with 0.05% Tween-20, and incubated with diluted HRP-conjugated antibodies specific for human IgG (1:10,000; AP004, Binding Site) for 3 h at room temperature. A chemiluminescent signal visualized by Chemi-Lumi One L (07,880–70, Nacalai Tesque) was detected with a ChemiDoc Touch Imaging System (Bio-Rad) and analyzed with Image Lab Software version 6.0.1 (Bio-Rad, https://www.bio-rad.com/en-jp/product/image-lab-software?ID=KRE6P5E8Z).

### Statistical analyses

All values are presented as the mean ± SEM. Antibody titers of < 1 and > 100,000 were assigned values of 1 and 100,000, respectively. The statistical significance of differences between two groups was assessed by Mann–Whitney U test. The correlation coefficient was calculated by Spearman’s rank correlation test. A difference was considered statistically significant when *p* < 0.05. Statistical analysis was performed using GraphPad Prism version 8.4.3 (GraphPad Software, https://www.graphpad.com/scientific-software/prism/).

### Study approval

This study was approved by the ethical committee of Osaka University Hospital (No. 19546). Residual serum samples were obtained following the completion of routine clinical laboratory testing. This study was also approved by the ethical committee of Osaka City University Hospital (No. 2020–110). In this study, the residual serum samples were used for the analysis without the patient’s information. Comprehensive informed consent has been previously obtained and approved in the ethical committee in Osaka City Hospital (No. 2927). Therefore, waiver of informed consent was approved for projects involving the secondary analysis of the residual serum samples, and we applied Opt-out method to obtain consent on this study by using the announcement of this study in web site. The experimental protocol including a statement of all methods were in accordance with the relevant guidelines and regulations by the ethical committee of Osaka University Hospital (No. 19546) and Osaka City University Hospital (No. 2020–110).

## Supplementary Information

Supplementary Information
