# Supplementary material for: SARS-CoV-2-induced humoral immunity through B cell epitope analysis in COVID-19 infected individuals
Source: Sci Rep. 2021 Mar 15;11:5934. doi: 10.1038/s41598-021-85202-9 (PMC7960719; doi:10.1038/s41598-021-85202-9)
Supplement: Supplementary file 1 — Supplementary Information [file 41598_2021_85202_MOESM1_ESM.pdf]

Supplemental Data

Table of Contents

Page 2: Supplemental Table 1. Candidate recombinant proteins for ELISA
Page 3,4: Supplemental Table 2. Top 20 strongest binding peptide regions of serum samples from each individual (OU #1-7)
Page 5: Supplemental Table 3. The serum titer against recombinant SARS-CoV-2 proteins and the neutralizing antibody titer of OU-COVID-19 patients
Page 6: Supplemental Figure 1. Anti-SARS-CoV-2 IgG subclass responses of COVID-19 patients.
Page 7: Supplemental Figure 2. Correlations between neutralizing activity and IgM antibody titer of COVID-19 patients.
Page 8: Supplemental Figure 3. The serum titer against recombinant SARS-CoV-2 nucleocapsid protein.

SARS-CoV-2-induced humoral immunity through B cell epitope analysis in COVID-19 infected individuals

Shota Yoshida,[1, 2] Chikako Ono [3], Hiroki Hayashi [1],
Shinya Fukumoto [4], Satoshi Shiraishi [5], Kazunori Tomono [6],
Hisashi Arase [7,8], Yoshiharu Matsuura [3], Hironori Nakagami [1]*

[1]Department of Health Development and Medicine, Osaka University Graduate School of Medicine.
[2]Department of Geriatric and General Medicine, Osaka University Graduate School of Medicine.
[3]Department of Molecular Virology, Research Institute for Microbial Diseases, Osaka University
[4]Department of Premier Preventive Medicine, Osaka City University School of Medicine
[5]Juso Osaka City.Hospital
[6]Division of Infection Control and Prevention, Osaka University Hospital
[7]Department of Immunochemistry, Researsch Institute for Microbial Diseases, Osaka University, Suita, Osaka 565-0871, Japan
[8]Laboratory of Immunochemistry, WPI Immunology Frontier Research Centre, Osaka University, Suita, Osaka 565-0871

Supplemental Table 1.

Candidate recombinant proteins for ELISA

Product	Manufacturer	Catalog Code	Expression System
SARS-CoV-2 (2019-nCoV) Spike S1-His Recombinant Protein	Sino Biological	40591-V08H	HEK293 Cells
SARS-CoV-2 (2019-nCoV) Spike RBD-Fc Recombinant Protein	Sino Biological	40592-V02H	HEK293 Cells
Recombinant 2019-nCoV/COVID-19 Spike S1 Protein (His Tag)	Beta Lifescience	BLPSN-0982P	HEK293 Cells
Recombinant 2019-nCoV Spike S1+S2 Protein (ECD, His tag)	Beta Lifescience	BLPSN-0986P	Baculovirus-Insect Cells
Recombinant 2019-nCoV Spike Protein S2 (ECD, His tag)	Beta Lifescience	BLPSN-0987P	Baculovirus-Insect Cells
Recombinant 2019-nCoV Spike Protein (RBD, His Tag)	Beta Lifescience	BLPSN-0988P	Baculovirus-Insect Cells
Recombinant SARS-CoV-2 Spike Protein, S1 Subunit	Ray Biotech	230-01101	Escherichia coli
Recombinant SARS-CoV-2 Spike Protein, S2 Subunit	Ray Biotech	230-01103	Escherichia coli

Supplemental Table 2.

Top 20 strongest binding peptide regions of serum samples from each individual (OU #1-7)

#1	Epitope ID	Type	Position	Amino Acid Sequence
1	A4	N	10 - 24	R-N-A-P-R-I-T-F-G-G-P-S-D-S-T
2	A5	N	13 - 27	P-R-I-T-F-G-G-P-S-D-S-T-G-S-N
3	A3	N	7 - 21	Q-N-Q-R-N-A-P-R-I-T-F-G-G-P-S
4	A2	N	4 - 18	N-G-P-Q-N-Q-R-N-A-P-R-I-T-F-G
5	F13	N	397 - 411	A-A-D-L-D-D-F-S-K-Q-L-Q-Q-S-M
6	C24	N	214 - 228	G-G-D-A-A-L-A-L-L-L-L-D-R-L-N
7	J23	M	211 - 225	S-S-S-S-D-N-I-A-L-L-V-Q
8	A12	N	34 - 48	G-A-R-S-K-Q-R-R-P-Q-G-L-P-N-N
9	A13	N	37 - 51	S-K-Q-R-R-P-Q-G-L-P-N-N-T-A-S
10	H3	M	7 - 21	T-I-T-V-E-E-L-K-K-L-L-E-Q-W-N
11	B18	N	124 - 138	G-A-N-K-D-G-I-I-W-V-A-T-E-G-A
12	D2	N	220 - 234	A-L-L-L-L-D-R-L-N-Q-L-E-S-K-M
13	C23	N	211 - 225	A-G-N-G-G-D-A-A-L-A-L-L-L-L-D
14	H2	M	4 - 18	S-N-G-T-I-T-V-E-E-L-K-K-L-L-E
15	J22	M	208 - 222	T-D-H-S-S-S-S-D-N-I-A-L-L-V-Q
16	F8	N	382 - 396	L-P-Q-R-Q-K-K-Q-Q-T-V-T-L-L-P
17	D1	N	217 - 231	A-A-L-A-L-L-L-L-D-R-L-N-Q-L-E
18	B19	N	127 - 141	K-D-G-I-I-W-V-A-T-E-G-A-L-N-T
19	F14	N	400 - 414	L-D-D-F-S-K-Q-L-Q-Q-S-M-S-S-A
20	B17	N	121 - 135	L-P-Y-G-A-N-K-D-G-I-I-W-V-A-T

#2	Epitope ID	Type	Position	Amino Acid Sequence
1	C1	N	145 - 159	H-I-G-T-R-N-P-A-N-N-A-A-I-V-L
2	D2	N	220 - 234	A-L-L-L-L-D-R-L-N-Q-L-E-S-K-M
3	C2	N	148 - 162	T-R-N-P-A-N-N-A-A-I-V-L-Q-L-P
4	D1	N	217 - 231	A-A-L-A-L-L-L-L-D-R-L-N-Q-L-E
5	A4	N	10 - 24	R-N-A-P-R-I-T-F-G-G-P-S-D-S-T
6	A6	N	16 - 30	T-F-G-G-P-S-D-S-T-G-S-N-Q-N-G
7	A5	N	13 - 27	P-R-I-T-F-G-G-P-S-D-S-T-G-S-N
8	J14	M	184 - 198	S-Q-R-V-A-G-D-S-G-F-A-A-Y-S-R
9	A7	N	19 - 33	G-P-S-D-S-T-G-S-N-Q-N-G-E-R-S
10	C5	N	157 - 171	I-V-L-Q-L-P-Q-G-T-T-L-P-K-G-F
11	D5	N	229 - 243	Q-L-E-S-K-M-S-G-K-G-Q-Q-Q-Q-G
12	J11	M	175 - 189	T-L-S-Y-Y-K-L-G-A-S-Q-R-V-A-G
13	A3	N	7 - 21	Q-N-Q-R-N-A-P-R-I-T-F-G-G-P-S
14	A2	N	4 - 18	N-G-P-Q-N-Q-R-N-A-P-R-I-T-F-G
15	J12	M	178 - 192	Y-Y-K-L-G-A-S-Q-R-V-A-G-D-S-G
16	D4	N	226 - 240	R-L-N-Q-L-E-S-K-M-S-G-K-G-Q-Q
17	D3	N	223 - 237	L-L-D-R-L-N-Q-L-E-S-K-M-S-G-K
18	H3	M	7 - 21	T-I-T-V-E-E-L-K-K-L-L-E-Q-W-N
19	J15	M	187 - 201	V-A-G-D-S-G-F-A-A-Y-S-R-Y-R-I
20	F1	N	361 - 375	K-T-F-P-P-T-E-P-K-K-D-K-K-K-K

#3	Epitope ID	Type	Position	Amino Acid Sequence
1	C24	N	214 - 228	G-G-D-A-A-L-A-L-L-L-L-D-R-L-N
2	C23	N	211 - 225	A-G-N-G-G-D-A-A-L-A-L-L-L-L-D
3	D1	N	217 - 231	A-A-L-A-L-L-L-L-D-R-L-N-Q-L-E
4	A22	N	64 - 78	L-K-F-P-R-G-Q-G-V-P-I-N-T-N-S
5	A3	N	7 - 21	Q-N-Q-R-N-A-P-R-I-T-F-G-G-P-S
6	D2	N	220 - 234	A-L-L-L-L-D-R-L-N-Q-L-E-S-K-M
7	A4	N	10 - 24	R-N-A-P-R-I-T-F-G-G-P-S-D-S-T
8	A2	N	4 - 18	N-G-P-Q-N-Q-R-N-A-P-R-I-T-F-G
9	A23	N	67 - 81	P-R-G-Q-G-V-P-I-N-T-N-S-S-P-D
10	C16	N	190 - 204	S-R-N-S-S-R-N-S-T-P-G-S-S-R-G
11	A24	N	70 - 84	Q-G-V-P-I-N-T-N-S-S-P-D-D-Q-I
12	B1	N	73 - 87	P-I-N-T-N-S-S-P-D-D-Q-I-G-Y-Y
13	A14	N	40 - 54	R-R-P-Q-G-L-P-N-N-T-A-S-W-F-T
14	C15	N	187 - 201	S-S-R-S-R-N-S-S-R-N-S-T-P-G-S
15	F2	N	364 - 378	P-P-T-E-P-K-K-D-K-K-K-K-A-D-E
16	C18	N	196 - 210	N-S-T-P-G-S-S-R-G-T-S-P-A-R-M
17	F1	N	361 - 375	K-T-F-P-P-T-E-P-K-K-D-K-K-K-K
18	A21	N	61 - 75	K-E-D-L-K-F-P-R-G-Q-G-V-P-I-N
19	D3	N	223 - 237	L-L-D-R-L-N-Q-L-E-S-K-M-S-G-K
20	F10	N	388 - 402	K-Q-Q-T-V-T-L-L-P-A-A-D-L-D-D

#4	Epitope ID	Type	Position	Amino Acid Sequence
1	A11	N	31 - 45	E-R-S-G-A-R-S-K-Q-R-R-P-Q-G-L
2	A12	N	34 - 48	G-A-R-S-K-Q-R-R-P-Q-G-L-P-N-N
3	A21	N	61 - 75	K-E-D-L-K-F-P-R-G-Q-G-V-P-I-N
4	C2	N	148 - 162	T-R-N-P-A-N-N-A-A-I-V-L-Q-L-P
5	A22	N	64 - 78	L-K-F-P-R-G-Q-G-V-P-I-N-T-N-S
6	B24	N	142 - 156	P-K-D-H-I-G-T-R-N-P-A-N-N-A-A
7	A20	N	58 - 72	Q-H-G-K-E-D-L-K-F-P-R-G-Q-G-V
8	C1	N	145 - 159	H-I-G-T-R-N-P-A-N-N-A-A-I-V-L
9	C7	N	163 - 177	Q-G-T-T-L-P-K-G-F-Y-A-E-G-S-R
10	A13	N	37 - 51	S-K-Q-R-R-P-Q-G-L-P-N-N-T-A-S
11	D2	N	220 - 234	A-L-L-L-L-D-R-L-N-Q-L-E-S-K-M
12	F5	N	373 - 387	K-K-K-A-D-E-T-Q-A-L-P-Q-R-Q-K
13	F6	N	376 - 390	A-D-E-T-Q-A-L-P-Q-R-Q-K-K-Q-Q
14	C4	N	154 - 168	N-A-A-I-V-L-Q-L-P-Q-G-T-T-L-P
15	C5	N	157 - 171	I-V-L-Q-L-P-Q-G-T-T-L-P-K-G-F
16	F10	N	388 - 402	K-Q-Q-T-V-T-L-L-P-A-A-D-L-D-D
17	C6	N	160 - 174	Q-L-P-Q-G-T-T-L-P-K-G-F-Y-A-E
18	F11	N	391 - 405	T-V-T-L-L-P-A-A-D-L-D-D-F-S-K
19	F13	N	397 - 411	A-A-D-L-D-D-F-S-K-Q-L-Q-Q-S-M
20	D10	N	244 - 258	Q-T-V-T-K-K-S-A-A-E-A-S-K-K-P

N, Nucleocapsid; M, Membrane; E, Envelope.

Supplemental Table 2.

Top 20 strongest binding peptide regions of serum samples from each individual (OU #1-7)

#5	Epitope ID	Type	Position	Amino Acid Sequence
1	D2	N	220 - 234	A-L-L-L-L-D-R-L-N-Q-L-E-S-K-M
2	C19	N	199 - 213	P-G-S-S-R-G-T-S-P-A-R-M-A-G-N
3	C18	N	196 - 210	N-S-T-P-G-S-S-R-G-T-S-P-A-R-M
4	D22	N	280 - 294	E-Q-T-Q-G-N-F-G-D-Q-E-L-I-R-Q
5	C17	N	193 - 207	S-S-R-N-S-T-P-G-S-S-R-G-T-S-P
6	B16	N	118 - 132	E-A-G-L-P-Y-G-A-N-K-D-G-I-I-W
7	D18	N	268 - 282	Y-N-V-T-Q-A-F-G-R-R-G-P-E-Q-T
8	B22	N	136 - 150	E-G-A-L-N-T-P-K-D-H-I-G-T-R-N
9	D3	N	223 - 237	L-L-D-R-L-N-Q-L-E-S-K-M-S-G-K
10	C23	N	211 - 225	A-G-N-G-G-D-A-A-L-A-L-L-L-L-D
11	C15	N	187 - 201	S-S-R-S-R-N-S-S-R-N-S-T-P-G-S
12	C24	N	214 - 228	G-G-D-A-A-L-A-L-L-L-L-D-R-L-N
13	B1	N	73 - 87	P-I-N-T-N-S-S-P-D-D-Q-I-G-Y-Y
14	J1	M	145 - 159	L-R-G-H-L-R-I-A-G-H-H-L-G-R-C
15	L3	E	7 - 21	E-E-T-G-T-L-I-V-N-S-V-L-L-F-L
16	I23	M	139 - 153	V-I-G-A-V-I-L-R-G-H-L-R-I-A-G
17	H1	M	1 - 15	M-A-D-S-N-G-T-I-T-V-E-E-L-K-K
18	E1	N	289 - 303	Q-E-L-I-R-Q-G-T-D-Y-K-H-W-P-Q
19	J2	M	148 - 162	H-L-R-I-A-G-H-H-L-G-R-C-D-I-K
20	D19	N	271 - 285	T-Q-A-F-G-R-R-G-P-E-Q-T-Q-G-N

#6	Epitope ID	Type	Position	Amino Acid Sequence
1	F16	N	406 - 420	Q-L-Q-Q-S-M-S-S-A-D-S-T-Q-A
2	J22	M	208 - 222	T-D-H-S-S-S-S-D-N-I-A-L-L-V-Q
3	F15	N	403 - 417	F-S-K-Q-L-Q-Q-S-M-S-S-A-D-S-T
4	F2	N	364 - 378	P-P-T-E-P-K-K-D-K-K-K-K-A-D-E
5	F17	N	409 - 423	Q-Q-S-M-S-S-A-D-S-T-Q-A
6	J21	M	205 - 219	K-L-N-T-D-H-S-S-S-S-D-N-I-A-L
7	A13	N	37 - 51	S-K-Q-R-R-P-Q-G-L-P-N-N-T-A-S
8	A12	N	34 - 48	G-A-R-S-K-Q-R-R-P-Q-G-L-P-N-N
9	A5	N	13 - 27	P-R-I-T-F-G-G-P-S-D-S-T-G-S-N
10	D2	N	220 - 234	A-L-L-L-L-D-R-L-N-Q-L-E-S-K-M
11	C21	N	205 - 219	T-S-P-A-R-M-A-G-N-G-G-D-A-A-L
12	A14	N	40 - 54	R-R-P-Q-G-L-P-N-N-T-A-S-W-F-T
13	E24	N	358 - 372	D-A-Y-K-T-F-P-P-T-E-P-K-K-D-K
14	J23	M	211 - 225	S-S-S-S-D-N-I-A-L-L-V-Q
15	B1	N	73 - 87	P-I-N-T-N-S-S-P-D-D-Q-I-G-Y-Y
16	C15	N	187 - 201	S-S-R-S-R-N-S-S-R-N-S-T-P-G-S
17	C14	N	184 - 198	S-R-S-S-S-R-S-R-N-S-S-R-N-S-T
18	C24	N	214 - 228	G-G-D-A-A-L-A-L-L-L-L-D-R-L-N
19	A11	N	31 - 45	E-R-S-G-A-R-S-K-Q-R-R-P-Q-G-L
20	F8	N	382 - 396	L-P-Q-R-Q-K-K-Q-Q-T-V-T-L-L-P

#7	Epitope ID	Type	Position	Amino Acid Sequence
1	A22	N	64 - 78	L-K-F-P-R-G-Q-G-V-P-I-N-T-N-S
2	A23	N	67 - 81	P-R-G-Q-G-V-P-I-N-T-N-S-S-P-D
3	C24	N	214 - 228	G-G-D-A-A-L-A-L-L-L-L-D-R-L-N
4	B24	N	142 - 156	P-K-D-H-I-G-T-R-N-P-A-N-N-A-A
5	F9	N	385 - 399	R-Q-K-K-Q-Q-T-V-T-L-L-P-A-A-D
6	A13	N	37 - 51	S-K-Q-R-R-P-Q-G-L-P-N-N-T-A-S
7	F10	N	388 - 402	K-Q-Q-T-V-T-L-L-P-A-A-D-L-D-D
8	B23	N	139 - 153	L-N-T-P-K-D-H-I-G-T-R-N-P-A-N
9	F13	N	397 - 411	A-A-D-L-D-D-F-S-K-Q-L-Q-Q-S-M
10	A20	N	58 - 72	Q-H-G-K-E-D-L-K-F-P-R-G-Q-G-V
11	A21	N	61 - 75	K-E-D-L-K-F-P-R-G-Q-G-V-P-I-N
12	A4	N	10 - 24	R-N-A-P-R-I-T-F-G-G-P-S-D-S-T
13	D10	N	244 - 258	Q-T-V-T-K-K-S-A-A-E-A-S-K-K-P
14	F8	N	382 - 396	L-P-Q-R-Q-K-K-Q-Q-T-V-T-L-L-P
15	D12	N	250 - 264	S-A-A-E-A-S-K-K-P-R-Q-K-R-T-A
16	F11	N	391 - 405	T-V-T-L-L-P-A-A-D-L-D-D-F-S-K
17	D1	N	217 - 231	A-A-L-A-L-L-L-L-D-R-L-N-Q-L-E
18	A3	N	7 - 21	Q-N-Q-R-N-A-P-R-I-T-F-G-G-P-S
19	H2	M	4 - 18	S-N-G-T-I-T-V-E-E-L-K-K-L-L-E
20	D3	N	223 - 237	L-L-D-R-L-N-Q-L-E-S-K-M-S-G-K

N, Nucleocapsid; M, Membrane; E, Envelope.

Supplemental Table 3.

The serum titer against recombinant SARS-CoV-2 proteins and the neutralizing antibody titer of OU-COVID-19 patients

Sample	Serum antibody titer (OD 50%)					Neutralization ID75
	S1+S2		RBD		Nucleocapsid	
	IgG	IgM	IgG	IgM	IgG	
OU #1	13.1	2.7	5.4	1	86.7	377.2
OU #2	24.1	1	32.3	1	3,441.4	10,864.4
OU #3	12.8	1	9.7	16.2	6,769.7	100,000
OU #4	1	1	1	1	5,124.8	9,540.9
OU #5	1	1	1	1	1	275.8
OU #6	461.0	1	11.3	1	1,508.3	100,000
OU #7	1	1	6.1	1	19.1	82.9
GMT	7.9	1.2	5.3	1.5	313.5	3,668.5

OU, serum samples collected from patients in the ICU of Osaka University Hospital.
ID75, 75% inhibitory dose
GMT, geometric mean titer.

Antibody titers of < 1 and > 100,000 were assigned values of 1 and 100,000, respectively.

Supplemental Figure 1.

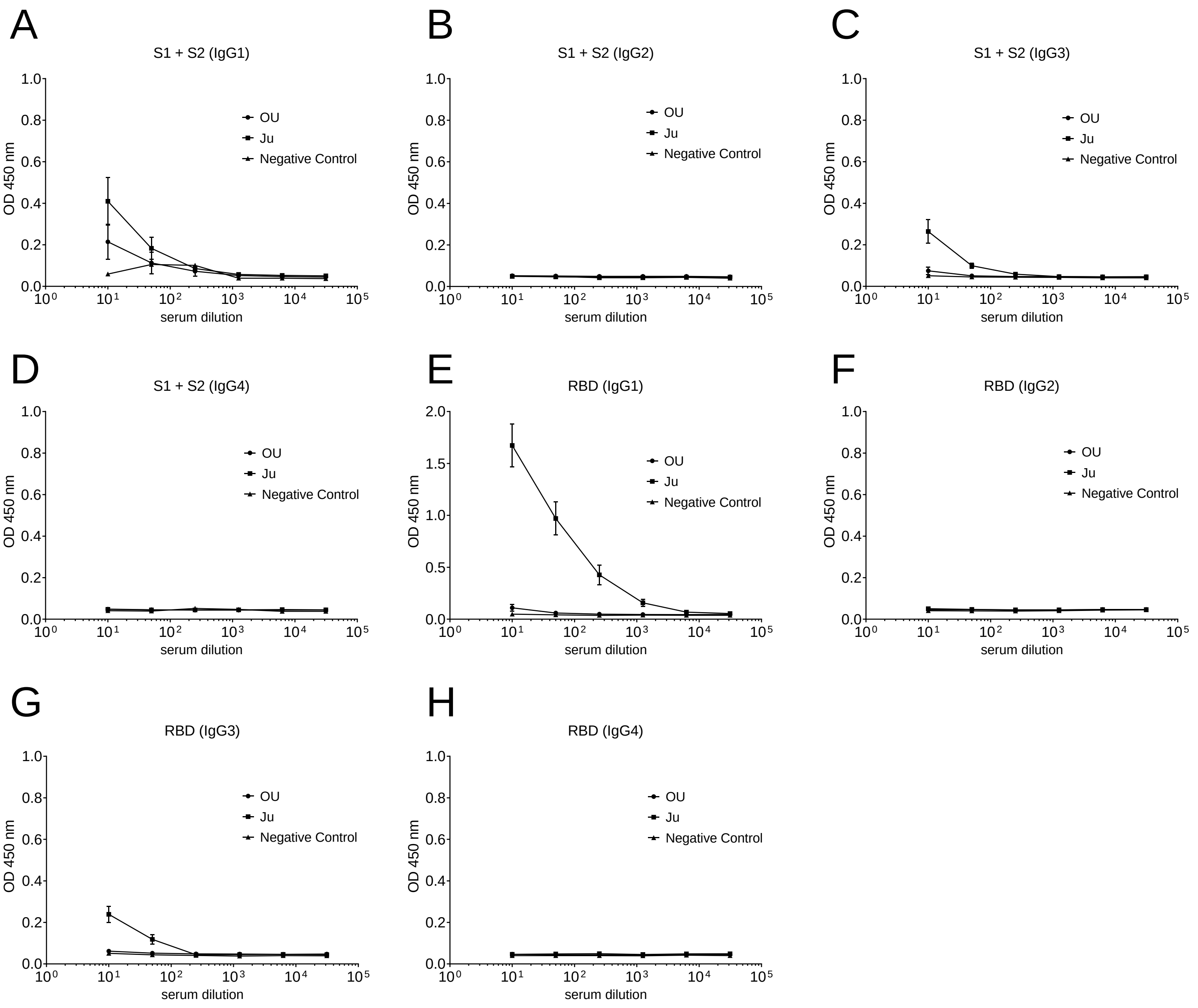

Anti-SARS-CoV-2 IgG subclass responses of COVID-19 patients.

(A-D) The serum titer against recombinant SARS-CoV-2 spike S1+S2 protein. **(A)** IgG1, **(B)** IgG2, **(C)** IgG3, **(D)** IgG4, expressed as the OD at 450 nm. **(E-H)** The serum titer against recombinant SARS-CoV-2 spike RBD protein. **(E)** IgG1, **(F)** IgG2, **(G)** IgG3, **(H)** IgG4, expressed as the OD at 450 nm. OU, serum samples collected from patients in the ICU of Osaka University Hospital (n = 7); Ju, serum samples collected from patients in Osaka City Juso Hospital (n = 6). All the data are expressed as the mean ± SEM. Graphs made in GraphPad Prism version 8.4.3, https://www.graphpad.com/scientific-software/prism/.

Supplemental Figure 2.

A

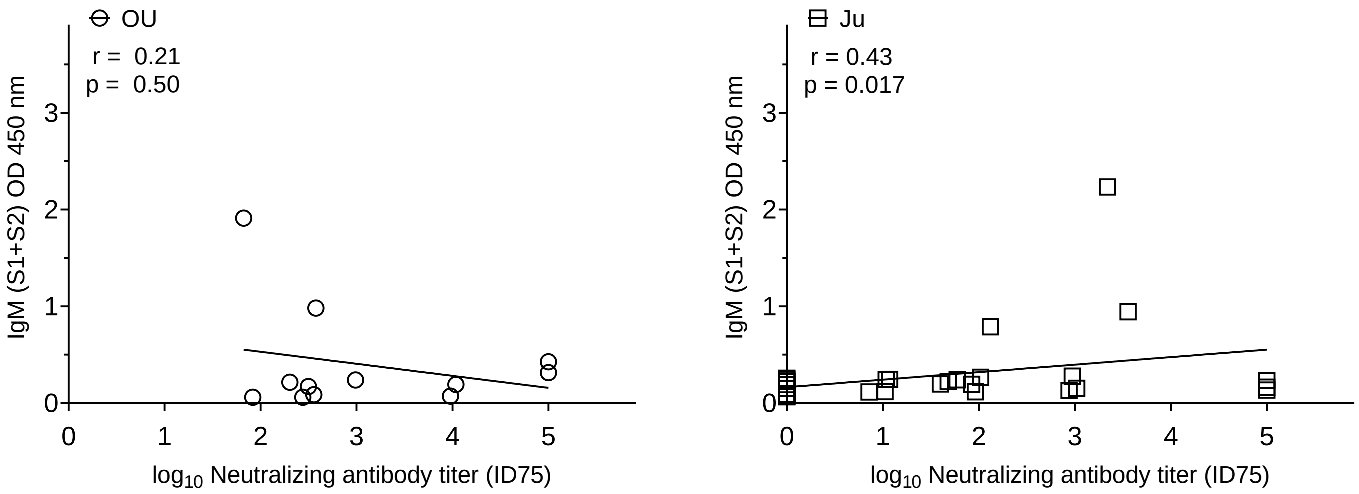

B

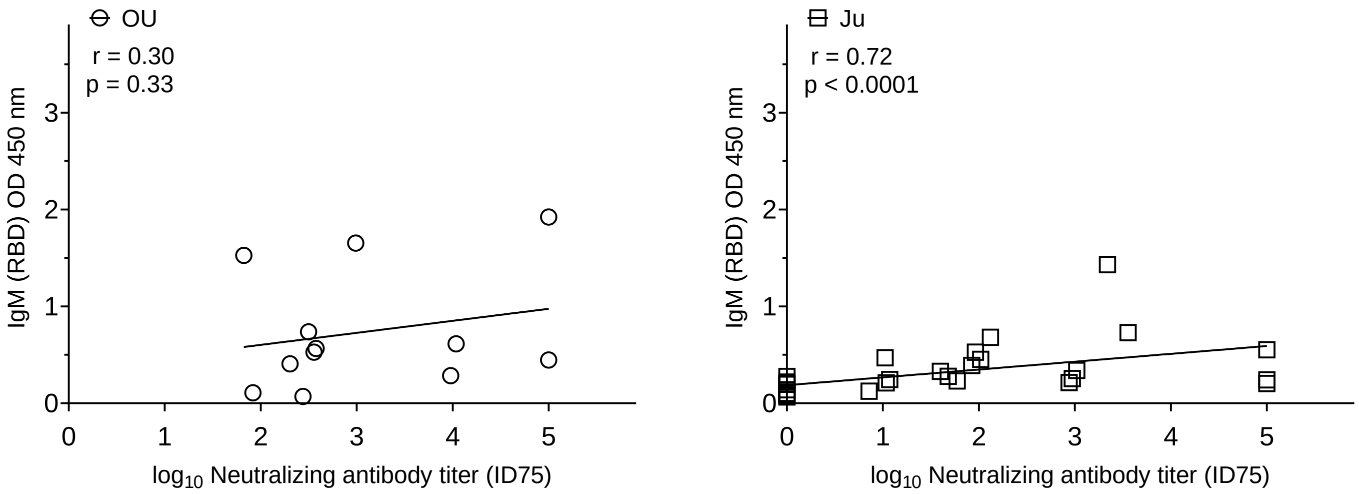

Correlations between neutralizing activity and IgM antibody titer of COVID-19 patients. **(A)** The correlations between neutralizing antibody titer (ID75) and anti-spike S1+S2 IgM titer (OD 450 nm, 10-fold dilution). **(B)** The correlations between neutralizing antibody titer (ID75) and anti-spike RBD IgM titer (OD 450 nm, 10-fold dilution). OU (left), serum samples collected from patients in the ICU of Osaka University Hospital (n = 12); Ju (right), serum samples collected from patients in Osaka City Juso Hospital (n = 31). The correlation coefficient was calculated by Spearman's rank correlation test, common logarithmic transformation (X axes). Graphs made in GraphPad Prism version 8.4.3, https://www.graphpad.com/scientific-software/prism/.

Supplemental Figure 3.

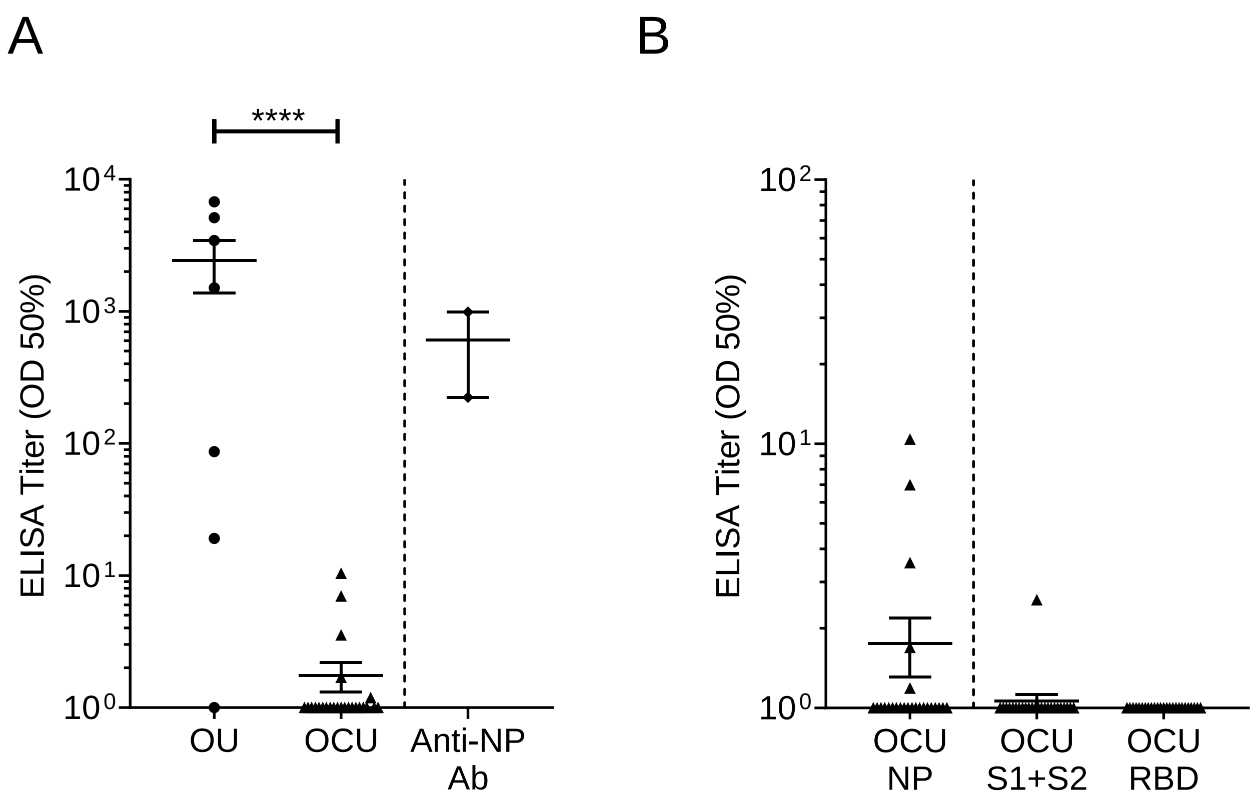

The serum titer against recombinant SARS-CoV-2 nucleocapsid protein.

(A) The serum IgG antibody titer against recombinant SARS-CoV-2 nucleocapsid protein (Acro Biosystems) is expressed as the half-maximal binding (OD 50%). **(B)** The serum IgG antibody titer against recombinant SARS-CoV-2 nucleocapsid protein (same data as (**A**) OCU), recombinant 2019-nCoV spike S1+S2 protein (Beta Lifescience) or recombinant 2019-nCoV spike protein (RBD; Beta Lifescience) is expressed as OD 50%. OU, serum samples collected from patients in the ICU of Osaka University Hospital (n = 7); OCU, serum samples collected from non-COVID-19 patients in Osaka City University Hospital in 2019 (n = 25); Anti-NP Ab, Anti-SARS-CoV-2 Nucleocapsid antibody (positive control, 1,000 µg/ml, Acro Biosystems). All the data are expressed as the mean ± SEM. Statistical evaluation was performed by Mann-Whitney U test; ****$p < 0.0001$. Graphs made in GraphPad Prism version 8.4.3, https://www.graphpad.com/scientific-software/prism/.
